# Disruption of respiratory epithelial basement membrane in COVID-19 patients

**DOI:** 10.1186/s43556-021-00031-6

**Published:** 2021-03-20

**Authors:** Xue Liu, Yinshan Fang, Paul W. Noble, Jianwen Que, Dianhua Jiang

**Affiliations:** 1 Department of Medicine, Division of Pulmonary and Critical Care Medicine, Women’s Guild Lung Institute, Cedars-Sinai Medical Center, Los Angeles, California USA; 2 Center for Human Development and Division of Digestive and Liver Disease, Department of Medicine, Columbia University Medical Center, New York, NY 10032 USA; 3 Department of Biomedical Sciences, Cedars-Sinai Medical Center, Los Angeles, California USA

Dear Editor,

Coronavirus SARS-CoV-2 has infected over 110 million people and the COVID-19 pandemic claimed more than 2.4 million lives worldwide as of February 17th, 2021. SARS-CoV-2 causes substantial pulmonary disease including pneumonia and acute respiratory distress syndrome (ARDS), especially in individuals at old age with multiple comorbidities and immunocompromisation [1]. The pathological features of COVID-19 lungs greatly resemble those seen in severe acute respiratory syndrome (SARS) and Middle Eastern respiratory syndrome (MERS) coronavirus infection. However, the pathogenesis of the disease is largely unclear.

Pathological findings showed the typical features of diffuse alveolar damage (DAD) in SARS-CoV-2 caused ARDS [2]. Lung biopsy of a COVID-19 patient showed bilateral DAD with desquamation of pneumocytes, hyaline membrane formation, and alveolar edema [2]. SARS-CoV-2 infected ciliated cells in the airway and type 2 pneumocytes (AT2) in alveolar regions [3]. Viral particles can be detected in AT2 cells and KRT5^+^ airway cells [4]. Notably, a significant depletion of AT1 and increased proliferation of AT2 cells were observed, suggesting that AT2 cells are mobilized to regenerate the damaged alveoli in COVID-19 lungs [4]. In the trachea and large airways, KRT5^+^ cells proliferated extensively, while in smaller airways predominant proliferating cells were lineage undetermined [4]. Interstitial mononuclear inflammatory infiltrates, dominated by lymphocytes, were seen in biopsied lungs [2]. Recently, an array of extrapulmonary manifestations have been reported in many organs and tissues [5]. Recent reviews also suggest viral sepsis in COVID-19 patients, as well as endothelial dysfunction and thrombotic microangiopathy [1]. We think that these extrapulmonary manifestations can be explained by the failure of the respiratory epithelial cells and their basement membrane. In this letter, by immunofluorescence co-staining, we examined the integrity of the respiratory epithelium, endothelium and the respiratory epithelial basement membrane, and discovered the loss of respiratory epithelial integrity and the epithelial basement membrane in COVID-19 patients.

The alveolar basement membrane is a critical component of blood–air barrier (BAB, or alveolar–capillary barrier) which prevents the formation of air bubbles in the blood, and from blood entering the alveoli. The alveolar basement membrane acts as scaffolds guiding morphogenesis, tissue repair, micromolecular permeability regulation, and cell movement. To demonstrate the structures and functions of the basement membranes in COVID-19 patients, we first examined COVID-19 lung sections from autopsies of three patients and three healthy donors (patient information was detailed in our previous study [4] and in supplemental materials). Immunoactivity of SARS-CoV-2 viral capsid spike (S) protein can be seen in the COVID-19 lung sections (Fig. 1a). Immunostaining for Laminin, the major component of basement membrane, on healthy lung sections gave clear and smooth basement membrane bands surrounding both alveoli and blood vessels (Fig. 1a, #1 and 2 areas). In COVID-19 lung sections, the alveolar and vascular structures were severely damaged, and the lumens were filled with nucleated and non-nucleated cells (Fig. 1a). Although positive Laminin staining could be detected in lung interstitial tissues, the bands were interrupted, and scattered Laminin protein was found in interstitial tissues. Moreover, fibrous Laminin was lining the alveolar-like structures in COVID-19 lung sections (Fig. 1a, area #3). Condensed cell areas were also identified (#4 area), suggesting fibrosis foci in COVID-19 lungs. Vascular-like round structures were found in these condensed cell areas (#3 and 4 areas) (Fig. 1a), suggesting vasculogenesis in those areas. These pathological changes can be found in all three cases of COVID-19 lungs (Supplementary Fig. 1).

**Fig. 1:**
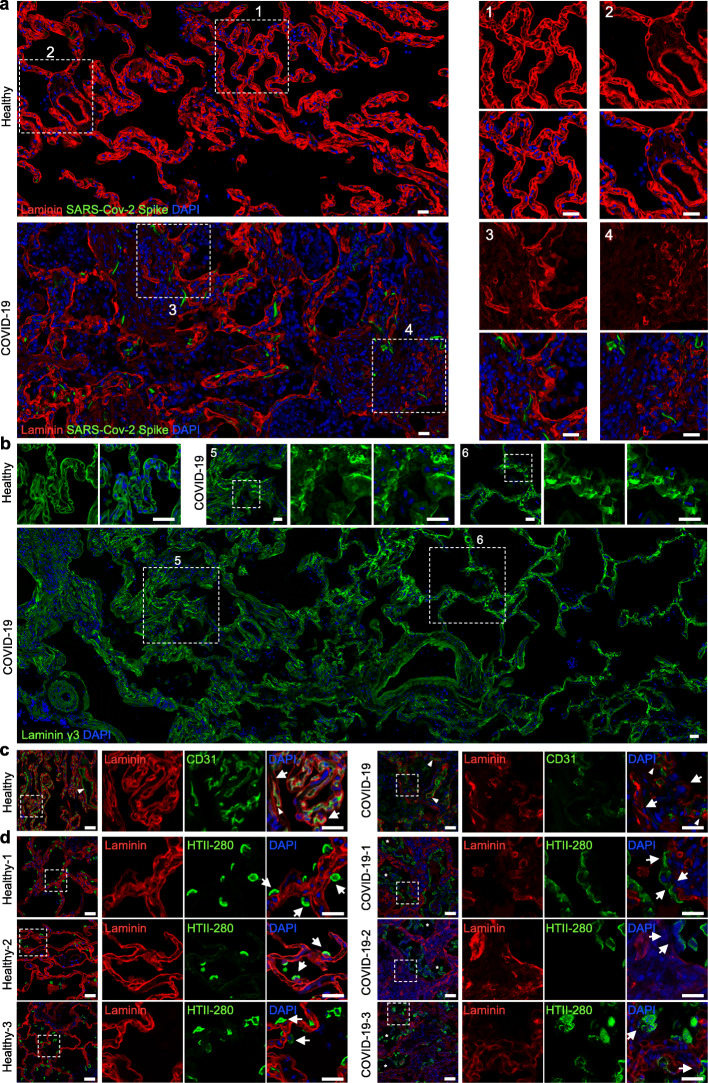
Disrupted alveolar epithelial basement membrane in COVID-19 patient lung. **a** Immunofluorescence for basement membrane marker, Laminin, and SARS-CoV-2 viral capsid protein “spike” on healthy and COVID-19 lung sections. Two boxed areas were magnified in both healthy and COVID-19 lung sections. **b** Laminin γ3 staining on healthy and COVID-19 lung sections. More and less severely damaged areas of COVID-19 patient lung areas were boxed and magnified on tile scanning images. **c** Co-staining of Laminin and endothelial cells marker, CD31, on healthy and COVID-19 lung sections. The Laminin-stained basement membranes were shattered (arrows). Note that the endothelium of both pulmonary vein and capillaries in COVID-19 lung was interrupted (arrowheads). **d** Co-staining for Laminin and AT2 marker, HTII-280, on healthy and COVID-19 lung sections. Clear Laminin^+^ bands were observed underneath AT2 cells in healthy lung but not in COVID-19 lung (arrows). Magnified areas are boxed. Asterisks, AT2 cells desquamated from alveolar walls. Scale bars, 20 μm

To further confirm epithelial basement membrane defects, we used another antibody to detect Laminin γ3 and observed similar staining structures in healthy lungs (Fig. 1b). In tile scanning COVID-19 sections, severely damaged lung structures and disrupted basement membrane bands were apparent. At higher magnifications, we found abnormal basement membrane structures in both severely damaged lung areas (#5 area) and less damaged areas (#6 area) (Fig. 1b). These observations were also confirmed in the other two COVOD-19 lungs.

To better locate the basement membrane, we co-stained the endothelial cell marker, CD31, with Laminin. In healthy lung sections, the endothelium was either well lined in blood vessels (arrowhead) or was evenly distributed in the alveolar walls (boxed area) and was surrounded by Laminin^+^ basement membrane (Fig. 1c, arrows). In COVID-19 lung sections, CD31 showed much less immunostaining signal in alveolar areas, and vascular endothelium was also disrupted (arrowheads). More importantly, the basement membrane was severely damaged (Fig. 1c, arrows).

We next examined AT2 cells and found dispersed HTII-280^+^ AT2 in healthy lung sections and smooth Laminin^+^ basement membrane right underneath AT2 cells (Fig. 1d, arrows). However, in COVID-19 sections, HTII-280^+^ cells were present in multiple cell clusters. Most AT2 cells were desquamated from the alveolar walls and were larger and more flattened compared to healthy AT2 cells (Fig. 1d, asterisks and arrows). The expression levels of laminin were lower in the alveolar walls and were barely detected in AT2 regions, suggesting damaged AT2 cell basement membrane (Fig. 1d). These pathological changes could be found in all three cases of COVID-19 lungs.

Taken together, we demonstrate that SARS-CoV-2 caused severe damage to the human lung. Cell-filled alveoli and condensed fibrosis-like cell regions are identified in COVID-19 lungs. The alveolar endothelium is severely damaged and the epithelial cell boundaries (BABs) are broken, leading to the inflow of cells and viral particles from the lung alveoli into blood. AT2 cells accumulate to form colonies and most cells are desquamated from the alveoli-like structures and exhibit altered cell morphologies. Most interestingly, the alveolar basement membranes, including epithelial and vascular basement membranes, are severely disrupted. Scattered and disconnected basement membrane components were identified in COVID-19 lungs. All these damages resembled the changes in alveolar and microvascular endothelial structure ARDS. We speculate that when the dams (the respiratory epithelial cells and their basement membrane) are breached, the flood (viral particles) can reach other organs, tissues, and cells which usually would not see the virus. Because of the heterogeneous nature of the “flood”, a wide array of extrapulmonary manifestations then present. These findings provide pathological insights in understanding the mechanisms of alveolar damage and alveolar repair. Further investigations into the underlying mechanisms of alveolar regeneration and basement membrane repair should help in developing strategies for combating lung damage as well as extrapulmonary injuries in COVID-19 patients. Also, in coordinate with the systemic inflammatory response induced “cytokine storm” and thromboembolism in COVID-19 patients, severity of basement membrane damage is likely to be a possible factor to clinical severity and mortality.

## Supplementary Information

**Additional file 1.** Supplementary Information for Materials and Methods. **Supplementary Fig.1.** Laminin staining on three COVID-19 patients and one normal lung sections.

## Data Availability

All data and material relevant to this publication is available upon reasonable request.

## Abbreviations

ARDS: Acute respiratory distress syndrome
SARS: Severe acute respiratory syndrome
MERS: Middle Eastern respiratory syndrome
DAD: Diffuse alveolar damage
AT2: Type 2 pneumocytes
AT1: Type 1 pneumocytes
BAB: Blood–air barrier
