# Supplementary material for: Disruption of respiratory epithelial basement membrane in COVID-19 patients
Source: Mol Biomed. 2021 Mar 20;2:8. doi: 10.1186/s43556-021-00031-6 (PMC7979449; doi:10.1186/s43556-021-00031-6)
Supplement: Supplementary file 1 — Additional file 1. Supplementary Information for Materials and Methods. Supplementary Fig.1. Laminin staining on three COVID-19 patients and one normal lung sections. [file 43556_2021_31_MOESM1_ESM.pdf]

Supplementary Materials for

Disruption of Respiratory Epithelial Basement Membrane in COVID-19 Patients

Xue Liu[1], Yinshan Fang[2], Paul W. Noble[1], Jianwen Que[2], Dianhua Jiang[1,3,*]

* Correspondence: Dianhua Jiang (Dianhua.Jiang@CSHS.org)

This PDF file includes:

Materials and Methods
Fig. S1

Materials and methods

Human specimens

The lung specimens of deceased COVID-19 patients were obtained from Biobank at Columbia University Irving Medical Center and lung samples of healthy donor were from Cedars-Sinai Medical Center. Lung sections from autopsies of three patients were from Biobank at Columbia University Irving Medical Center and healthy lung sections from three healthy donors were from Cedars-Sinai Medical Center. The patient information was previously reported [1]. Brief information of healthy donors and COVID-19 patients was: healthy donors, male, 62 years old (sample # CC002-20) male, 67 years old (sample # CC001-15), and male 18 years old (sample # CC007-19); COVID-19 patients, male, 65 years old (sample # 144471), male, 72 years old (sample # 144538), and female, 68 years old (sample # 144511).

Histology and immunostaining experiments

The lung tissues were fixed in 4% paraformaldehyde at 4℃ overnight. Tissues for frozen sections were incubated in 30% sucrose and 30% sucrose + Tissue-Tek® O.C.T. compound 1:1 mixture overnight, respectively, and then were embedded in O.C.T. compound (Sakura) and sectioned at 10 µm for immunofluorescence staining. Tissues for paraffin sections were dehydrated through a series of grade ethanol, cleared by xylene and then embedded in paraffin. 5 µm sections were cut and mounted on the glass slides for further staining. Paraffin sections were dewaxed and rehydrated, and antigen retrieval was performed in citric acid-based antigen unmasking solution by high-pressure heating. Cryosections were rehydrated by being incubated in PBS twice. Rehydrated sections were blocked with 5% normal goat serum (Kumar, 2014 #18) (Jackson ImmunoResearch) and incubated in diluted primary antibodies overnight at 4℃. Primary antibodies used were: rabbit anti-Laminin polyclonal antibody (Novus Biologicals, NB300-144, RRID AB_10001146, 1:100), rabbit anti-Laminin gamma 3 monoclonal antibody (abcam, ab234429, 1:200), mouse anti-human-CD31 monoclonal antibody (Agilent Dako, M082329-2, 1:50), mouse anti-HTII-180 IgM monoclonal antibody (a gift from L. Dobbs lab of UCSF, 1:100). Secondary antibodies used were: Cy™3 Donkey Anti-Rabbit IgG (H+L) (Jackson ImmunoResearch, 711-165-152, RRID AB_2307443), Alexa Fluor® 488 Donkey Anti-Rabbit IgG (H+L) (Jackson ImmunoResearch, 711-545-152, RRID AB_2313584), Alexa Fluor® 488 Goat Anti-Mouse IgG (H+L) (Jackson ImmunoResearch, 115-545-146, RRID AB_2307324), Goat anti-Mouse IgG / IgM (H+L) Secondary Antibody, Alexa Fluor 488 (ThermoFisher Scientific, A-10680, RRID AB_2534062). All the secondary antibodies were diluted in 5% NGS at 1:200. Secondary antibodies incubation was performed at room temperature for 1.5 hours protected from light. For the monoclonal Anti-SARS-CoV S Protein spike (Similar to 240C) (BEI Resources, NR-616, 1:100) antibody staining on cryosection, biotinylated anti-mouse IgG antibody (Vector laboratories, BA-9200, RRID AB_2336171, 1:100), ABC-HRP reagent R.T.U. peroxidase (Vector laboratories, PK-7100, RRID AB_2336827) and TSA fluorescein (Perkin Elmer, SAT701001KT, 1:400) were used. The slides were mounted in VECTASHIELD Antifade Mounting Medium with DAPI (Vector Labs) and photographed with a Zeiss LSM 780 confocal.

Reference

1 Fang Y, Liu H, Huang H *et al.* Distinct stem/progenitor cells proliferate to regenerate the trachea, intrapulmonary airways and alveoli in COVID-19 patients. *Cell Res* 2020; **30**:705-707.

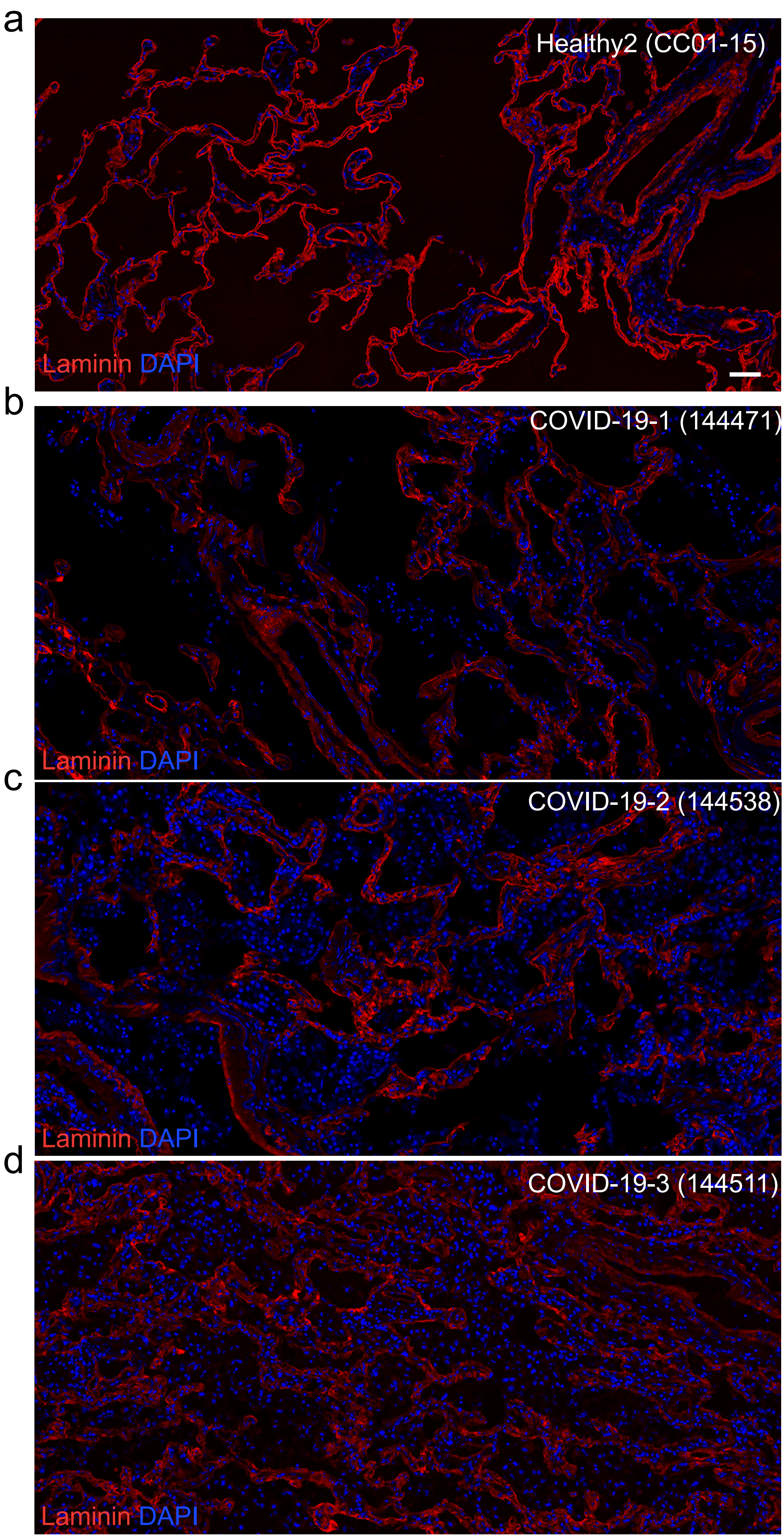

Fig. S1. Laminin staining on three COVID-19 patients and one normal lung sections. Representative tile scanning images of laminin-stained lung sections from one normal (**a**) and three COVID-19 patients and COVID-19 patient (**b**-**d**) sections. Scale bar, 50 μm.
